# Connecting reservoir computing with statistical forecasting and deep neural networks

**DOI:** 10.1038/s41467-021-27715-5

**Published:** 2022-01-11

**Authors:** Lina Jaurigue, Kathy Lüdge

**Affiliations:** 1 Technische Universität Berlin, Institut für Theoretische Physik, Hardenbergstraße 36, 10623 Berlin, Germany; 2 Technische Universität Ilmenau, Institut für Physik, Weimarer Straße 25, 98693 Ilmenau, Germany

**Keywords:** Mathematics and computing, Other photonics, Electronics, photonics and device physics

## Abstract

Among the existing machine learning frameworks, reservoir computing demonstrates fast and low-cost training, and its suitability for implementation in various physical systems. This Comment reports on how aspects of reservoir computing can be applied to classical forecasting methods to accelerate the learning process, and highlights a new approach that makes the hardware implementation of traditional machine learning algorithms practicable in electronic and photonic systems.

The prediction of the future development of an a priori unknown and complex system is a task that can be tackled by algorithms that are based on unconventional (or analog) computing. There are a wide range of classical regression models that have been developed for, or applied to, the task of time-series forecasting with different levels of complexity, e.g. from the statistical forecasting field there are classical linear methods such as autoregressive integrated moving-average methods (ARIMA) or vector-autoregression (VAR), and extensions of these methods such as nonlinear vector-autoregression (NVAR). From the machine learning field, there is reservoir computing (RC) on the lower end of the complexity spectrum, deep neural networks (DNN) on the upper end, and various other learning algorithms in between. The machine learning algorithms are also suited to solving a range of other tasks in addition to time-series forecastings, such as picture or speech recognition.

In this comment, we want to draw attention to recent studies published in Nature Communications^1,2^, which connect elements of this hierarchy of methods and facilitate the transfer of knowledge between research fields.

The connection between the different methods discussed here is RC. We will first explain its concept and briefly compare it with DNN and NVAR. RC was first introduced by H. Jaeger under the name of the ‘echo-state‘ machine^3^. It consists of a recurrent network (the reservoir) that is driven by input data and output is produced by linearly combining the state of the readout nodes, as illustrated in Fig. 1b. For traditional neural network algorithms, all weights are trained, as depicted in Fig. 1a for a deep neural network. In contrast to this, only the output weights are trained in RC (yellow lines in Fig. 1b), via linear regression. This reduction in the complexity of the training has to be compensated for by an increase in the dimension of the reservoir, but it means that any physical dynamical system can be used as a reservoir (e.g. biological tissues, buckets of water, or semiconductor networks)^4^. The idea behind RC is that the reservoir performs nonlinear transforms on the input and that, if the network is appropriately chosen and the readout sampled correctly, the desired output can be approximated. In methods such as NVAR, the nonlinear transforms of the input are chosen directly and make up the so-called feature vector, the elements of which are linearly combined to produce the output (see Fig. 1c). A certain similarity between RC and NVAR is apparent and it was recently shown that there are conditions under which these methods are equivalent^5^.

**Fig. 1:**
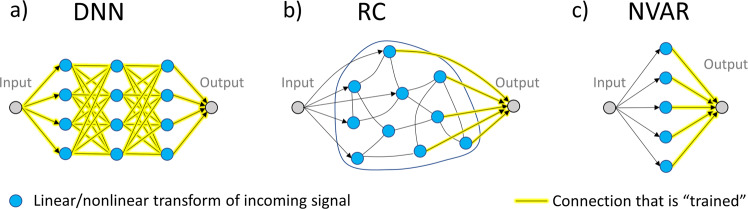
Visualization of different machine learning architectures. Topology and training scheme for the three different concepts discussed in this comment. DNN deep neural networks; RC reservoir computing, NVAR nonlinear vector-auto-regression.

Inspired by the results of Bollt^5^ the authors of Gauthier et al. ^1^ applied aspects of RC to NVAR and thereby introduced what they call *Next Generation Reservoir Computing* (NG-RC). Specifically, Tikhonov regularization is used and the role of correlations in the feature vector is also considered. With their approach, the authors achieve good results for typical time-series prediction tasks, while simultaneously having several advantages compared with conventional RC. Firstly, the absence of a reservoir means that there are fewer hyperparameters to tune. Secondly, the authors show that, at least for their chosen tasks, shorter training data sets are required and that the dimension of the output vector is smaller than the number of nodes for comparable reservoir computers. These two factors also lead to shorter computation times. If similar reductions in the required training data sets are also viable for real-world problems, where training data is often limited, then NG-RC could indeed be favorable. However, using this approach essentially trades the optimization of reservoir hyperparameters for the optimization of the elements of the feature vector, and the latter is still very much an open problem. It remains to be seen if the choice of the feature vector is generally an easier process than hyperparameter optimization for reservoir computers.

Although the authors of Gauthier et al. ^1^ refer to their method as RC, they also state that it most closely resembles NARX and it could be argued that it is in fact also a statistical learning method. However, one important difference between machine learning in general and statistical methods is the intended purpose^6^. For NARX methods the selection algorithms are designed such that only a few terms are selected and the resulting model is compact and transparent enough to gain insights into the relationship between elements of the underlying system^7^. Whereas, in machine learning, the goal is exclusively the optimization of the performance for a given task. The latter approach is also taken for the NG-RC method and its feature vectors have far more terms than for typical NARX methods. It is the difference in the choice of feature vector terms and the use of Tikhonov regularization that sets the NG-RC apart from the well-established statistical methods. The authors of^1^ do, however, address that the number of components in the feature vector can possibly be reduced without significantly influencing the error, as some of the components are very small. In this regard, NG-RC could be of interest to the statistical forecasting community, if it is possible to reduce the resulting NG-RC to a manageable model from which inferences about the underlying system can be made.

A lot of the interest in RC, in recent years, stems from the possibility for hardware implementation, as there are substantial gains to be made in terms of speed and power consumption compared with the implementation on a traditional computer^4^. Particularly suited to this is the concept of delay-based RC which was introduced in ref. ^8^ (see Fig. 2a). In this case, the reservoir need only consist of one nonlinear element with time-delayed self-feedback. For small inputs, the RC performance can be deduced from the linear response of the physical node^9^. Adding delay to any system makes it technically infinitely dimensional. In practical terms the systems do not have infinite dimensions, however, if the parameters are chosen correctly, such a system can exhibit complex, high-dimensional transient dynamics and can therefore perform well on various machine learning benchmarking tasks, see for example^10^. Using just a single node with a delay instead of a network of randomly coupled nodes is a great simplification that makes this scheme especially suited for hardware implementation with optical devices^11^.

**Fig. 2:**
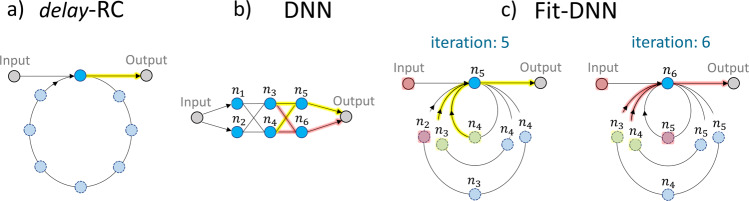
Deep neural networks via time delay and sequential sampling. Illustration of **a** delay-based RC scheme with a delay loop and one physical node (blue circle), **b** deep neural network (DNN) with three layers (each layer contains two physical nodes), and **c** corresponding folded-in-time deep neural network (Fit-DNN) realized via three feedback loops with time-varying feedback strengths and sequential sampling of the physical node (iteration 5 and 6 are shown).

In^2^ the authors take this idea of using only a single physical node with delay and extend it to emulate deep neural networks, an approach which they have coined *Folded-in-time Deep Neural Network* (Fit-DNN). This is achieved by having multiple delay loops with adjustable feedback strengths, while the physical node supplies the nonlinearity (see Fig. 2c for the Fit-DNN with the corresponding DNN in Fig. 2b). A network node of the Fit-DNN is now defined as the system state at a certain time and all nodes are sampled sequentially. Coupling between the layers is achieved by coupling back the appropriate time-delayed signals. The authors show that if the temporal separation between the nodes, i.e. the time intervals at which the system is sampled, is sufficiently large compared with the characteristic timescales of the physical node, then the Fit-DNN is equivalent to a deep neural network. When the node separation is small, there are additional inter-layer and intra-layer connections because temporally adjacent nodes are not fully independent, and a modified back-propagation method needs to be used to train the system. For small node separation and for the case of a sparse DNN, i.e. a Fit-DNN with a reduced number of delay loops, the authors test the performance on various benchmark tasks. For the sparse Fit-DNN they find good performance, but emphasis that removing/adding delay loops changes an entire diagonal of a coupling weight matrix and for this, a new training method is still required. In the case of small node separation, the performance is diminished, however, this needs to be weighed up with the decreased computation time that can be achieved by reducing the time between the sampling of the nodes. For a fully connected Fit-DNN with large node separation, the conventional DNN is fully reproduced with unaltered performance.

Overall, we find the novel method introduced in ref. ^2^ very interesting and hope to see hardware implementations of this approach in the near future. However, at this point, we must also mention a significant drawback that the authors also discuss. Although this approach is well suited for implementation in hardware, for example in photonic or optoelectronic setups, the training must still be performed using a conventional computer. Furthermore, due to the need to solve a delay differential equation, the training time can be significantly increased. Therefore the speed and efficiency of the final trained system need to be weighed up against the training process.

To summarize, the two newly introduced non-conventional computing schemes, i.e. NG-RC and Fit-DNN, suggest ways to realize effective and high-performing machine learning applications with small ecological footprints. Furthermore, bringing together knowledge from different communities, here the statistical learning, the nonlinear dynamics, and the machine learning communities, has led to cross-fertilization with high innovative potential.
